# Editorial: From Sequence to Functional Interpretation: Sifting Through the Variation of Genomic Data

**DOI:** 10.3389/fgene.2022.932299

**Published:** 2022-06-17

**Authors:** Andrea Ciolfi, Viviana Caputo, Daniel C. Koboldt, Paolo Uva

**Affiliations:** ^1^ Genetics and Rare Diseases Research Division, Bambino Gesù Children’s Hospital, IRCCS, Rome, Italy; ^2^ Department of Experimental Medicine, Sapienza University of Rome, Rome, Italy; ^3^ Institute for Genomic Medicine at Nationwide Children’s Hospital, Columbus, OH, United States; ^4^ IRCCS Istituto Giannina Gaslini, Genoa, Italy

**Keywords:** genomic variation, variant prioritization, splicing, mtDNA, whole genome sequencing

The study of natural genome variation in humans with Mendelian diseases has been an effective strategy to link genotype to phenotype. Following the success of whole exome sequencing (WES), which has made it possible to explain the molecular causes underlying about one-third of affected individuals, whole genome sequencing (WGS) offers an appealing strategy to address the additional complexity of genome structure. However, after excluding protein coding regions, canonical splice sites, or structural variants overlapping known disease-associated genes, WGS has thus far provided a modest improvement in diagnostic rates, mostly by reducing the search space *via* linkage analysis, homozygosity mapping, or identification of shared chromosomal microdeletions or duplications. Hence, new *in silico* methods are needed to identify different classes of variants and prioritize them at the whole-genome scale.

WES/WGS experiments are producing a wealth of information about human genome variation, including single/multiple (SNV/MNV) nucleotide variants, insertions/deletions, and more complex structural variants. However, the identification and interpretation of all these variants can be challenging, since “general-purpose” algorithms developed to date in most cases lack sensitivity for specific classes of variation that could underlie Mendelian traits. Efficient variant prioritization requires the integration of different tools and various levels of annotations. The goal of this Research Topic is to extend prioritization strategies for genomic data to interpret the functional impact of variants that do not show any effect on protein coding regions, *e.g.*, those in regulatory elements, splicing regions, UTRs, imprinted regions, simple tandem repeats, or the mitochondrial genome.

Even though variants affecting splicing are predicted to account for up to 15% of molecular causes in Mendelian disorders, recent evidence suggests that variation outside canonical splice sites could be underestimated. The role of some types of splicing variants—such as deep intronic variants whose effect on splicing is challenging to predict based on location and sequence context—is particularly difficult to interpret. Therefore, new strategies are needed to effectively predict their clinical relevance.

To address this issue, Ohno and collaborators (Takeda et al.) took advantage of a new *in silico* tool to refine the prediction of intronic variants affecting splicing. To predict the pathogenicity of SNVs at intronic positions (from −50 to −3), the authors developed gradient boosting-based machine learning models (LightGBM) trained on well-established pathogenic SNVs and population polymorphisms. In this paper, the authors showed that the new LightGBM model outperformed previous algorithms used for the same scope in all statistical measures of accuracy and precision, providing a new useful resource to evaluate SNV impact on splicing.

Another approach is to consider the variant impact on splicing, as carried out by Chen and collaborators (Qian et al.) who developed a strategy to investigate the contribution of deep-intronic splice variants to inherited retinal diseases (IRDs) by combining a recently developed *in silico* tool with an *in vitro* minigene assay, to further validate the effect of these variants. Through this study, the authors defined a framework for deep intronic variant prioritization and estimated that the contribution of deep-intronic splice mutations to IRD patients is significantly under-appreciated.

NGS has also been widely applied to identify variants in mitochondrial DNA (mtDNA), which have been implicated in a broad spectrum of disorders with different degrees of penetrance. Analysis of mtDNA requires the adaptation of software originally developed for nuclear DNA and the development of novel approaches to take into account the specific features of mtDNA such as a large number of copies and heteroplasmy (*i.e.*, the presence of different genotypes within the same cell). In their work, Ip et al. assessed the accuracy of multiple variant callers using synthetic data with known heteroplasmic and homoplasmic variants. The authors reported consistent results across callers for homoplasmic variants, while a low concordance was observed for heteroplasmic variant calling, therefore caution should be taken when analyzing heteroplasmic variants, in particular at low allele frequencies.

Analysis of mtDNA is even more challenging for ancient DNA (aDNA) due to degradation and contamination with exogenous mtDNA from modern humans or other species. Reconstruction of mtDNA genome from aDNA and the assessment of heteroplasmy were the aims of the work of Diroma and others (Diroma et al.), who developed a computational pipeline for ancient mtDNA analysis. The pipeline includes steps for detection of contaminants, realignment of circular genomes, variant calling and filtering, haplogroup detection, and assembly of consensus sequences. The workflow has been successfully applied to both real and simulated data.

Understanding the functional effect of variants implicated in disease by genome-wide association studies is a major goal but also a challenging task, as most variants on current SNP arrays are in noncoding regions. To address this issue, it is crucial to consider the biological effect of DNA variants in specific cell types and tissues to properly evaluate the mechanistic links between genetic associations and human traits. As several context-free and context-specific computational prediction methods for this purpose are currently available, Torshizi et al. compare the effectiveness of both approaches to prioritize candidate causal variants. Their work highlights the differences between algorithm performance and suggests that they may play complementary roles. The Authors introduce a comparative multistep pipeline that was used in a proof-of-concept study on schizophrenia to provide a map of schizophrenia risk loci for experimental functional analyses.

The critical task of variant interpretation remains a major priority in human genetics, particularly as the paradigm for genomic studies shifts to WGS. The studies collected for this Research Topic showcase the diversity of methods and strategies that hold promise for meeting this challenge in the years to come.

